# Supplementary material for: Unhealthful plant-based diet associates with frailty risk predominantly in men with low income from the UK Biobank cohort
Source: J Nutr Health Aging. 2024 Dec 20;29(3):100463. doi: 10.1016/j.jnha.2024.100463 (PMC12179992; doi:10.1016/j.jnha.2024.100463)
Supplement: Supplementary file 2 [file mmc2.docx]

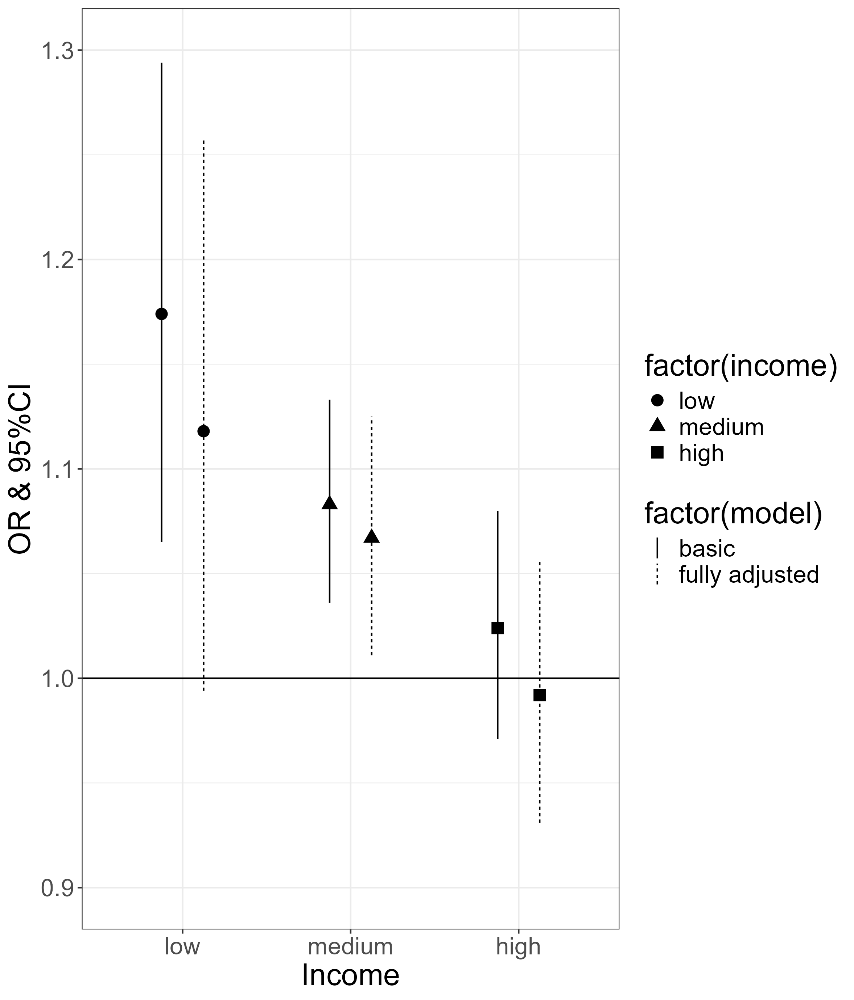


**Suppl. Figure 1 Association between unhealthful plant-based diet adherence and risk of frailty among men of different income groups after adjusting for atypical dietary intake**

OR per 10-unit increase in uPDI

− Basic model --- fully adjusted model ● low income ▲ medium income ■ high income


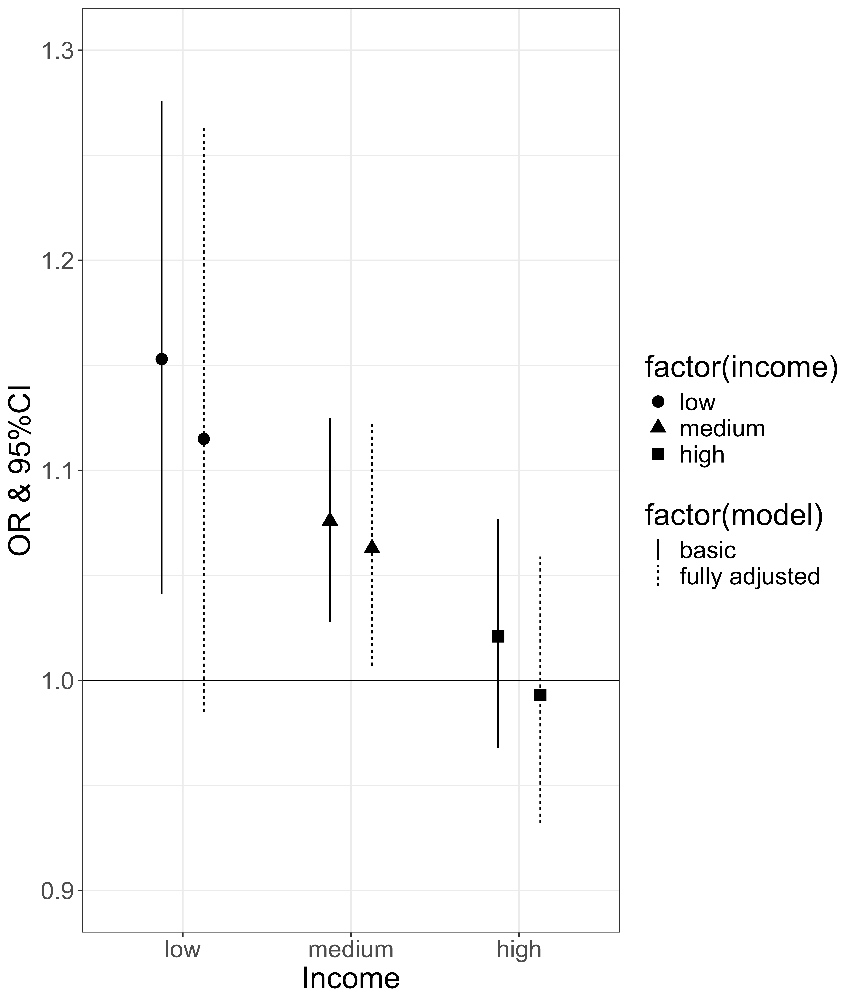


**Suppl. Figure 2 Association between unhealthful plant-based diet adherence and risk of frailty among men of different income groups assessed by binary logistic regression**

OR per 10-unit increase in uPDI

− Basic model --- fully adjusted model ● low income ▲ medium income ■ high income


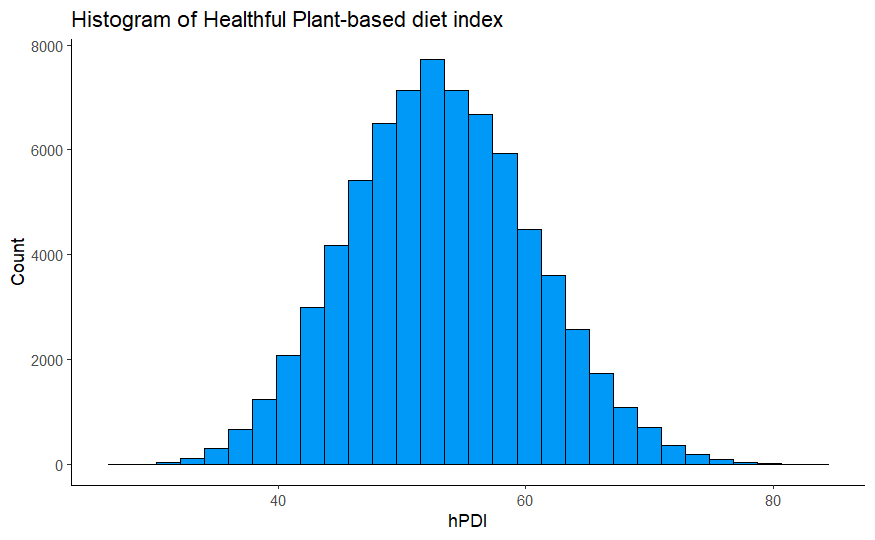


**Suppl. Fig 3: Histogram showing the distribution of the healthful plant-based diet index in the Study population**


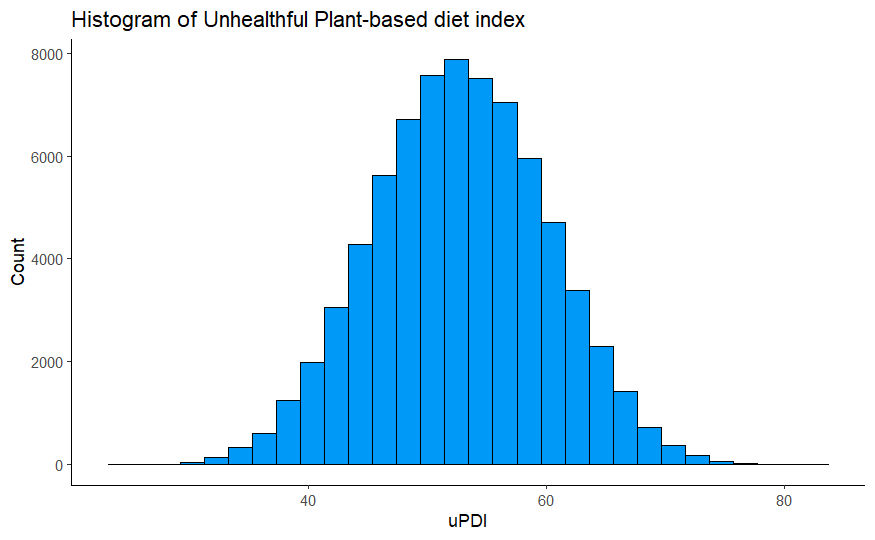


**Suppl. Fig 4: Histogram showing the distribution of the unhealthful plant-based diet index in the Study population**
